# Long-Term Administration of Queen Bee Acid (QBA) to Rodents Reduces Anxiety-Like Behavior, Promotes Neuronal Health and Improves Body Composition

**DOI:** 10.3390/nu10010013

**Published:** 2017-12-23

**Authors:** Michael J. Weiser, Vivian Grimshaw, Kelly M. Wynalda, M. Hasan Mohajeri, Christopher M. Butt

**Affiliations:** 1 Translational Biology, DSM Nutritional Products, 4909 Nautilus Court North, Suite 230, Boulder, CO 80301, USA; mike.weiser@dsm.com (M.J.W.); vivian.grimshaw@dsm.com (V.G.); kelly.wynalda@dsm.com (K.M.W.); 2 Biological Models, DSM Nutritional Products, Wurmisweg 576, Bld. 205/216, CH 4303 Kaiseraugst, Switzerland; hasan.mohajeri@dsm.com

**Keywords:** queen bee acid, neuroprotection, behavior, mood, body composition

## Abstract

Background: Queen bee acid (QBA; 10-hydroxy-2-decenoic acid) is the predominant fatty acid in royal jelly (RJ) and has activity at estrogen receptors, which affect brain function and body composition. However, few, long-term studies have assessed QBA effects in brain health and body composition. Methods: Primary hippocampal neurons were treated with QBA (0–30 µM) and challenged with glutamate or hypoxia. QBA was fed to aged, male Sprague-Dawley rats (12–24 mg/kg/day) and to adult male and female Balb/C mice (30–60 mg/kg/day) for ≥3.5 months. Rats were evaluated in a behavioral test battery of brain function. Mice were measured for fat and muscle composition, as well as bone density. Results: QBA increased neuron growth and protected against glutamate challenge and hypoxia challenge. Rats receiving QBA had reduced anxiety-like behavior, increased body weight, and better maintenance of body weight with age. Mice receiving QBA exhibited increased body weight, muscle mass, and adiposity in males, and increased bone density, but decreased adiposity, in females. Conclusions: QBA is an active component of RJ that promotes the growth and protection of neurons, reduces anxiety-like phenotypes, and benefits bone, muscle and adipose tissues in a sex-dependent manner, which further implicates estrogen receptors in the effects of QBA.

## 1. Introduction

Queen bee acid (QBA; 10-hydroxy-2-decenoic acid), is the predominant fatty acid constituent of royal jelly (RJ), and RJ is harvested from a select population of honeycomb cells in a beehive that are designed specifically for queen bee larvae. Royal jelly is believed to play a role in the enhanced size, fertility, and lifespan of queen bees as it contains a complex mixture of nutrients. The functions of this mixture are not understood completely [1,2,3]. Early reports indicated that RJ increased the growth of ovaries in rats [4], and anecdotal evidence in humans suggests that RJ benefits fertility, prolongs life, enhances skin quality and mood, and fights cancer. Some of these claims are supported by limited clinical evidence. Six-month ingestion of RJ (3 g/day; *N* = 30–31 per arm) in human males and females increased erythropoiesis, glucose tolerance, and testosterone levels. Mental health, as reported on self-assessments of general health, was also improved [5]. When humans consumed RJ for two weeks (10 g/day; *N* = 49; not placebo controlled), no adverse effects occurred and significant improvements in mood and cholesterol were noted in individuals that were older than 60 years of age [6]. Similarly, use of RJ (6 g/day for 4 weeks) by individuals that were approximately 40 years old (*N* = 7) resulted in lower cholesterol levels when compared to age-matched individuals who received no supplementation (*N* = 8) [7]. These findings demonstrate that RJ is likely safe, but the clinical assessments of RJ’s efficacy have clearly not been robust. Many of the RJ publications suggest that various components of RJ may be responsible for the effects that were observed, one of which could be QBA (see the above as well as Elnagar, 2010; Hashimoto et al., 2005; Hattori et al., 2011; Husein and Haddad, 2006; Karaca et al., 2010; Mishima et al., 2005; Nagai et al., 2006; Nakaya et al., 2007; Taniguchi et al., 2003) [8,9,10,11,12,13,14,15,16].

Approximately 2–6% of the total wet weight of RJ is QBA, and QBA comprises ~40% of the total fatty acids found in RJ ([1,17]. QBA inhibits histone deacetylases, and this mechanism is recognized as the epigenetic switch for the queen bee larvae phenotype [2]. Other in vitro effects of QBA include the stimulation of neurogenesis from stem cells while inhibiting gliogenesis [18]. In vivo administration of QBA (100–500 µg/kg/day injected intraperitoneally for 21 days) decreased anxiety-like and depressive-like behavior in young mice (49–70 days old) that were exposed to stress. However, QBA had no effect in unstressed animals [19]. Extracellular signal-regulated kinase (erk) signaling that induces the expression of brain derived neurotrophic factor (BDNF) has been proposed as a mechanism for the in vitro and in vivo effects of QBA. However, 10-carbon, monounsaturated fatty acids do not require the receptors of BDNF and the other neurotrophins for their downstream erk-related activities [20]. Low concentrations of QBA (100 pM–10 µM) also modulate the estrogen receptor function without interfering with estradiol binding [21,22]. Interestingly, non-genomic estrogen receptor activity results in downstream erk signaling as well as the induction of at least three other intracellular signaling cascades [23]. These findings suggest that QBA is an active component of RJ, a stressor may be necessary to detect its effects on mood, and modulation of erk signaling by the interactions between QBA and estrogen receptors is possible.

Two other issues exist with our current knowledge of QBA. First, an efficacious and commercially viable dose has not been determined. Second, the potential benefits of long-term, oral administration of QBA to healthy individuals have not been evaluated. In an attempt to address these issues, we performed concentration-response studies of neuroprotection and growth in primary rat hippocampal neurons in vitro, translated their findings into an in vivo behavioral test battery of mood and cognition in aged rats, and monitored the rats’ body weights over a five month exposure to QBA (0, 12, or 24 mg/kg/day). These studies tested the hypothesis that if QBA was beneficial to the central nervous system (CNS), then QBA would promote growth or neuroprotection in vitro, confer improvements in mood- or cognition-related behaviors in vivo, and would not have adverse effects on body weight. The results suggest that QBA has brain health benefits during normal development and aging, and that it may combat age-related and stress-related weight loss. However, body composition studies in mice indicated that the types of body weight changes were sex dependent, further implicating estrogen receptors as important sites of QBA activity.

## 2. Materials and Methods 

### 2.1. Animals

For all of the in vitro studies, female, timed-pregnant Sprague-Dawley rats (60–120 days old) were obtained from Envigo, Inc. (Indianapolis, IN, USA), and brain tissue was derived from their E17 pups after Caesarean section. For the primary in vivo studies, adult male Sprague-Dawley rats (12 months of age) were obtained from Envigo, were housed in pairs at the animal facilities of the University of Colorado, Boulder, and were maintained on a 12 h:12 h light-dark schedule with ad libitum access to chow and water. Similarly, for body composition studies, male and female Bagg albino (BALB/c) mice (60 days of age; Jackson Laboratory, Bar Harbor, ME, USA) were housed in groups of five at the animal facilities of the University of Colorado, Boulder, and were maintained on a 12 h:12 h light-dark schedule with ad libitum access to chow and water. All of the animal protocols were approved by the Institutional Animal Care and Use Committees at Bolder BioPATH (Boulder, CO, USA; in vitro; reference #BBP-002; approved July 2011, reviewed yearly, and re-approved 27JUL2017) and at the University of Colorado, Boulder (in vivo; reference #2383; approved 26MAY2015, reviewed yearly, and re-approved 23FEB2017).

### 2.2. Neurite Extension

Primary hippocampal neurons were isolated from day (E17–18) rat pups using a modified version of previously described protocols [24,25] that select for neuronal growth over glial growth [26]. Viable cell number and concentration were determined by trypan blue exclusion and were counted using an automated cell counter (Countess, Invitrogen, Carlsbad, CA, USA). Cell suspensions were diluted in warm Neurobasal/B27 media (Invitrogen) and plated at a density of 30,000 cells per well in 96-well plates (Falcon Optilux plates, BD Biosciences, Bedford, MA, USA) coated with laminin (Sigma-Aldrich, St. Louis, MO, USA) and poly-d-lysine (Sigma-Aldrich,). Upon plating, the cells were incubated with QBA (0–30 µM) for 7 days, with half of the media being removed and replaced with fresh media every other day, while maintaining the concentrations of the QBA treatments. For all of the in vitro methods, QBA was dissolved in methanol, and the final methanol concentration in the treatments did not exceed 0.1% by volume. Methanol vehicle controls were matched to each QBA concentration that was used, and these controls did not differ significantly from each other in any in vitro measure. The cultures were then stained with the neuron-specific marker, microtubule associated protein-2a (Map2a; MAP378 Millipore, Billerica, MA, USA), and cell nuclei were stained with 4′,6-diamidino-2-phenylindole (DAPI; Sigma-Aldrich). Fluorescent images of the staining were then captured with confocal microscopy (Olympus Fluoview 1000; Waltham, MA, USA). The staining was quantitated by dividing the percent area of the Map2a staining by the number of nuclei, and all of the cells were Map2a-positive.

### 2.3. Hypoxia Challenge

As described above, hippocampal neurons were derived from E17–18 pups and incubated for seven days. The neurons were pretreated with QBA (0–30 µM) for 48 h and subsequently exposed to hypoxia (<0.3% O_2_) for 48 h. The cultures were stained with calcein AM (Invitrogen), a marker of live cells, with ethidium homodimer (Sigma-Aldrich), a marker of cell death, and with tetramethylrhodamine ethyl ester (TMRE; Sigma-Aldrich), a marker for mitochondrial health [27]. The fluorescence of each of these markers was then quantitated with a fluorimeter.

### 2.4. Glutamate Challenge

As described above, hippocampal neurons were derived from E17–18 pups and incubated for 7 days. The cells were pretreated with QBA (0–30 µM) for 48 h and subsequently challenged with glutamate (25 µM) for 24 h. As with the hypoxia cultures, the cells were then stained with calcein AM, ethidium homodimer, and TMRE, and the fluorescence of each marker was quantitated with a fluorimeter.

### 2.5. Diets

The in vivo diets were designed to provide 0, 12 or 24 mg/kg/day of QBA (Cayman Chemical, Ann Arbor, MI, USA) to the rats or an equivalent QBA dose to the mice (0, 30 or 60 mg/kg/day; [24]). The diets were based on the American Institute of Nutrition (AIN)-93G standard, but their fat contents consisted of controlled blends of coconut oil, high-oleic safflower oil, and olive oil (Dyets, Inc.; Bethlehem, PA, USA). The lipid profiles of the oils were determined prior to diet formulation, and the lipid profiles of the diets were similarly determined after formulation. As a percentage of total fatty acids, each diet contained 51% saturates, 46% monosaturates, 7% linoleic acid, 0.25% linolenic acid, and no arachidonic, docosahexaenoic or eicosapentaenoic acids, with less than 0.5% variance in these parameters between diets. Thus, all of the fatty acids were balanced between diets except for QBA content. The diets were fed for 3.5 months prior to the behavioral studies, and the animals were maintained on the defined diets until all in vivo work was complete. 

### 2.6. Open Field Test

After three months of exposure to QBA and at 15 months of age, adult male rats were tested in the open field arena. The rats were also handled each day for the four days directly prior to evaluating open field activity. Handling involved gently removing the animal from its cage and holding it in the experimenter’s lap on a towel without restraint, allowing the animal to explore. This procedure acclimatized the animal to handling by experimenters. All the behavioral testing occurred in a room adjacent to the colony room, and the animals were acclimated to the test room overnight prior to testing. Each rat was placed in the middle of the circular chamber (100 cm diameter × 40 cm tall; 1200 lux), and behavior in the open field was recorded for 30 min with a digital camera and measured by AnyMaze software (5.1, Stoelting Co., Inc., Wood Dale, IL, USA). The total distance traveled and the number of entries into the center zone were then acquired. Entry into the center zone was defined as 75% of the animal entering the central, concentric area of the arena that had a diameter of 50 cm.

### 2.7. Barnes Maze

Spatial learning and memory performance was tested at age 16–17 months, and after 4–5 months of QBA exposure, using the Barnes circular maze. The maze consisted of a 122-cm diameter, gray circular platform raised 90 cm above the floor level with 20 holes, 10 cm in diameter, evenly spaced around the circumference. One hole fed into an escape tunnel beneath it, the three remaining training positions that were 90°, 180°, and 270° from the escape tunnel were equipped with dummy covers, and all of the other holes remained open. The animals were then trained to locate the escape tunnel beneath one of the four training positions in response to an aversive light stimulus (1200 lux).

The first day of training was used for acclimation to the maze. Each animal was placed in the center of the maze in a random direction. The animal was allowed to explore the maze for a maximum of 2 min. If the animal found the escape tunnel on its own, then it was allowed to remain in the tunnel for 1 min. If it did not find the escape tunnel within the 2 min, it was gently guided towards and into the escape tunnel, and allowed to remain in the tunnel for 1 min. The tunnel position varied between rats, and the maze was cleaned with a 70% ethanol solution between each session.

The next four days after acclimation were used for task acquisition training. The rats underwent two trials per day with a 3-min inter-trial interval. Again, each animal was started by placing them in the center of the maze in a random direction, the tunnel training position was varied between rats, and the maze was cleaned with a 70% ethanol solution between each session. In addition, the escape tunnel training position was at least 90° away from its acclimation position for each rat. Each trial lasted a maximum of 5 min, and the trials ended early if the animal reached the escape tunnel. Upon entering the tunnel, the animal was allowed to stay for 1 min before being returned to its homecage. If an animal did not find the tunnel by the end of a trial, it was removed from the maze and was returned to its homecage. The latency to enter the escape tunnel in each of two trials across four trial days was recorded with AnyMaze software.

Two weeks after the last training trial, the rats were tested on memory retention with a single, 5-min probe trial. The position of the target hole was the same as during the training period. However, the target hole now also had a dummy cover. The AnyMaze software then monitored how much time was spent within 15 cm of the target, the distance from the target, the number of memory mistakes at incorrect holes, and the amount of time spent at incorrect holes. All of the other parameters and cleaning between animals applied.

### 2.8. Elevated Plus Maze

Testing of the aged male rats (17–18 months) on the elevated plus-maze occurred after 5–6 months of QBA exposure. The plus maze was elevated 85 cm above the floor and consisted of a central platform (10 × 10 cm) that was connected to two open and two closed arms of the same size (50 cm long × 10 cm wide) that radiate to form a plus sign. The closed arms were surrounded by walls that were 40 cm high (Lafayette Instruments, Lafayette, IN, USA), and the ambient lighting was maintained at 150 lux. Each rat was placed in the central platform facing one of the open arms, and its behavior was recorded during a 5-min testing period with AnyMaze software. The amount of time spent on open and closed arms and the number of entries into open and closed arms were then assessed. Entry was defined as 75% of the animal being in the arm. Stretch-attends were defined as the animal being bent in a 90-degree orientation and 50% of the animal outside of a given arm. Head dips were defined as the animal being on the open arm, and its head dipping completely below the plane of the arm.

### 2.9. Forced Swim Test

Depressive-like behavior was assessed at age 18 months, and after six months of QBA exposure, using the forced swim test. The test chambers were cylindrical Plexiglas containers (45 cm high × 20 cm diameter; Stoelting) filled with 30 cm of fresh water (25 °C). The test was performed over two consecutive days. On day 1, the animals were acclimated to the test (08:00 h to 13:00 h) by placing them in a filled chamber for 15 min, after which they were toweled dried and returned to their home cage. On day 2 (24 h later) the test was performed for a total swim time of 5 min, after which the rats were toweled dried and returned to their home cage. Both trials were recorded by a digital video camera secured to the ceiling above the cylinders. Total time swimming, immobile, and climbing, and the number of dives were measured post hoc by an experimenter blind to the group assignments. Swimming was defined as movement of the forelimbs and hind limbs that did not break the surface of the water. Immobility was defined as absence of any movement except for slight movements necessary for the animal to keep its head above water. Climbing was defined as rapid movement of the forelimbs that broke the surface of the water. 

### 2.10. Weight Maintenance

As part of the in vivo work described above, the aged male rats were weighed on a weekly basis, and their food consumption (per cage mate pair) was monitored three times per week for the first two months of dietary supplementation.

### 2.11. Body Composition

In an effort to understand any weight effects, male and female BALB/c mice, two months of age, were fed QBA at doses (0, 30 or 60 mg/kg/day) that were equivalent to those given the rats [28]. Mice were used in these experiments because they allowed for an increased sample size and increased sampling frequency by way of their size compatibility with the available instruments. The food consumption of the mice was monitored throughout the feeding period. In addition, every seven days the mice were weighed, and the amounts of adipose, muscle and non-adipose tissues were determined with a tabletop time-domain-NMR spectrometer (Bruker Minispec Mq; Billerica, MA, USA). Each NMR assessment required less than 30 s to complete, each mouse was held motionless inside the spectrometer with a polystyrene plug equipped with ventilation holes, and anesthesia was not required. 

After four months of QBA exposure, final measurements of weight and NMR body composition were taken, and the mice were euthanized by exposure to 4% isoflurane gas followed by 100% CO_2_. The carcasses were then used directly for bone density analysis by peripheral quantitative computed tomography (pQCT; Stratec XCT Research SA+; Pforzheim, Germany). Trabecular bone density for each animal was determined by the average of three scans of the distal femoral metaphysis. The scans were taken at 1.50, 2.25 and 3.00 mm proximal to the distal edge of the femur. Cortical bone density for each animal was determined by a single scan of the tibial diaphysis at 50% of the length of the tibia (voxel size of 70 µm for all measurements) [29,30]. The parameters for trabecular bone analysis were contour mode = 2, peel mode = 2, and inner threshold = 700 mg/cm^3^. The parameters for cortical bone analysis were separation mode = 2 and threshold = 700 mg/cm^3^.

### 2.12. Statistical Analysis

Group sizes were determined by power analysis of preliminary data for each outcome measure (Minitab 17, ; Minitab, Inc., State College, PA, USA). The data were graphed using GraphPad Prism 6 and were analyzed using GraphPad 6 (GraphPad, Inc.; San Diego, CA, USA) or SPSS 23.0 (IBM; Armonk, NY, USA). Food consumption and data from the elevated plus maze, forced swim test, and pQCT were compared by using 1-way ANOVA, followed by Tukey’s post hoc test. Activity in the open field arena was summed into 10-min bins and analyzed by 1-way repeated measures ANOVA (RMANOVA) followed by Tukey’s post hoc test. Escape latencies in the Barnes maze and body weights and compositions over time were also analyzed by 1-way RMANOVA, followed by Tukey’s post hoc test. Statistical significance was set at α levels (*p*) less than 0.05.

## 3. Results

### 3.1. Neuron Growth

**Figure 1:**
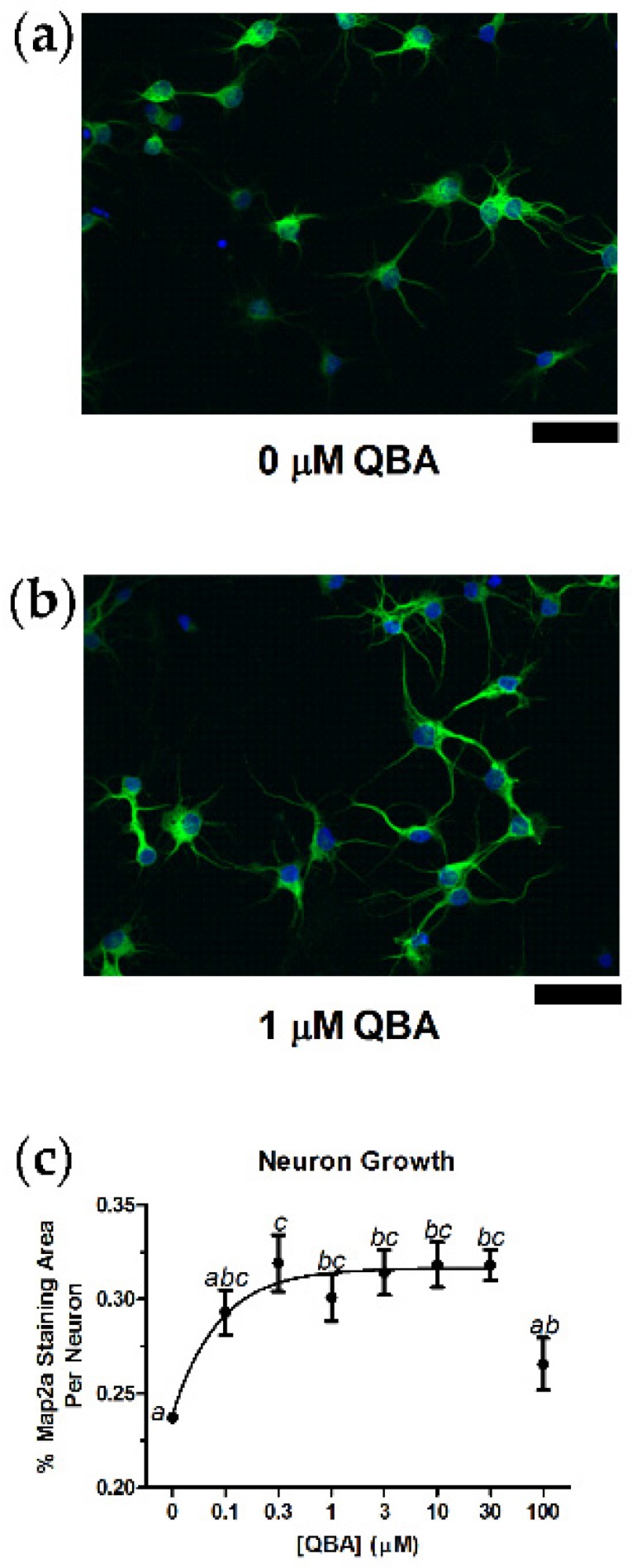
Queen bee acid (QBA) increased hippocampal neuron growth. The extent of growth was assessed by dividing the percent area of the Map2a fluorescence (green) by the number of neuronal nuclei identified by the DAPI signal (blue). (**a**) Control neurons were qualitatively smaller and less connected than neurons provided QBA (1 µM) for seven days (**b**). (**c**) Concentration-response curve for QBA effects on neuron growth. Lack of shared superscripts indicates data points that differed significantly (*p* < 0.05) in Tukey’s post hoc test. *N* = 5–6 in triplicate over two separate culture runs. Error bars represent the standard error of the mean (SEM). Scale bars = 50 µm.

QBA increased the growth of primary hippocampal neurons. Neurons grown in the absence of QBA (Figure 1a) appeared smaller, had shorter neurites and exhibited less connectivity than neurons grown in the presence of QBA (Figure 1b). A concentration-response curve was constructed after the percent area of Map2a immunocytochemical staining was quantified and normalized to each neuron in a given visual field. The half-maximal effective concentration (EC_50_) of QBA for neuron growth was 75.9 ± 29.1 nM, and maximal growth occurred between 0.3–30 µM (Figure 1c). Treatment with QBA concentrations above 30 µM resulted in a reduction from maximal growth, but a significant, overall treatment effect was detected (F_(7,39)_ = 5.88, *p* < 0.001).

### 3.2. Hypoxia Challenge and Glutamate Challenge

**Figure 2:**
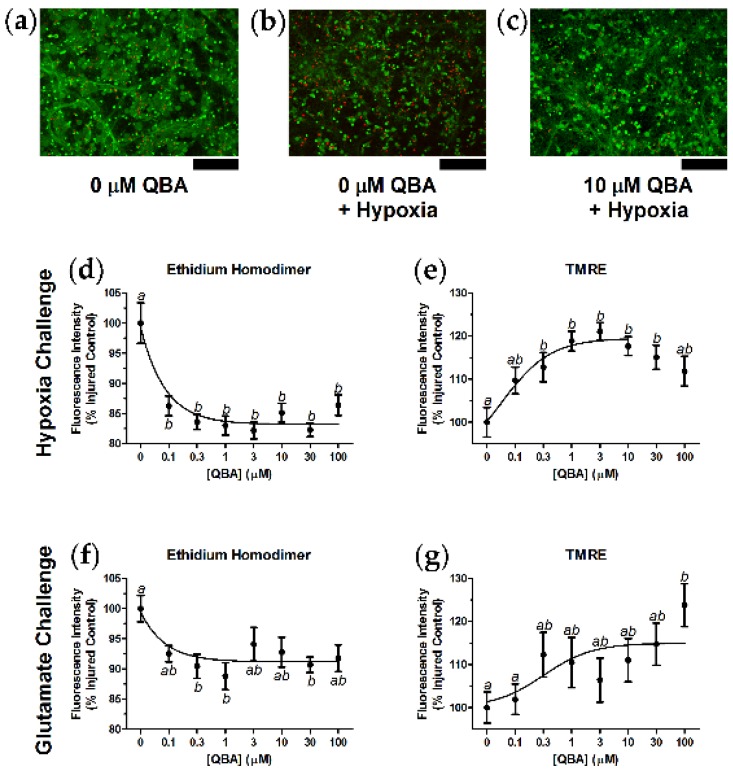
QBA decreased neuron death and increased mitochondrial health after hypoxia challenge and after glutamate challenge. (**a**) Control cells had qualitatively greater calcein AM staining (green), a marker of live cells, and less ethidium homodimer staining (red), a marker for dead cells, than (**b**) neurons challenged with hypoxic conditions (<0.3% O_2_ for 48 h). (**c**) The addition of QBA (10 µM) to hypoxic conditions reduced the number of dead cells. Similar results with QBA addition were observed in the presence of glutamate (25 µM) challenge (not shown). (**d**) Quantitation of ethidium homodimer fluorescence indicated that QBA decreased hypoxia-induced cell death by approximately 15%. (**e**) Conversely, separate experiments with TMRE, a marker of healthy, properly polarized mitochondria, demonstrated that QBA increased mitochondrial health in hypoxic neurons by 15–20%. (**f**) Concentration-response curve for QBA effects on glutamate-induced cell death, and (**g**) concentration-response curve for QBA effects on mitochondrial health during glutamate challenge. Lack of shared superscripts indicates data points that differed significantly (*p* < 0.05) in Tukey’s post hoc test. *N* = 13–16 in triplicate over two separate culture runs. Error bars represent the SEM. Scale bars = 100 µm.

QBA reduced mortality and increased mitochondrial health in primary hippocampal neurons that were exposed to either hypoxia or to glutamate challenge. Control neurons grown in the absence of both QBA and hypoxia (Figure 2a) had greater calcein AM fluorescence (live cell marker) and less ethidium homodimer fluorescence (dead cell marker) than control neurons exposed to hypoxic conditions (Figure 2b). The addition of QBA to the hypoxia challenge reduced the amount of cell death (Figure 2c), and similar qualitative findings were seen in separate experiments using glutamate challenge (not shown). QBA significantly reduced hypoxia-induced neuronal death (F_(7,105)_ = 9.26, *p* < 0.001) with a half-maximal inhibitory concentration (IC_50_) of 64.6 ± 17.9 nM (Figure 2d). Figure 2e illustrates that experiments with TMRE, a marker of properly polarized mitochondria, also indicated that QBA significantly increased mitochondrial health in hypoxia-challenged neurons (F_(7,107)_ = 5.14, *p* < 0.001), with an EC_50_ of 156.9 ± 58.4 nM.

The protective effects of QBA during glutamate challenge were less robust than during hypoxia challenge, but the glutamate-related QBA effects were still significant. Queen bee acid inhibited glutamate-induced neuronal death (F_(7,116)_ = 2.52, *p* = 0.019) with an IC_50_ of 56.0 ± 27.3 nM (Figure 2f). Accordingly, QBA increased mitochondrial health in glutamate-challenged neurons (F_(7,115)_ = 2.26, *p* = 0.034) with an EC_50_ of 317.5 ± 200.3 nM (Figure 2g).

### 3.3. Behavior

**Figure 3:**
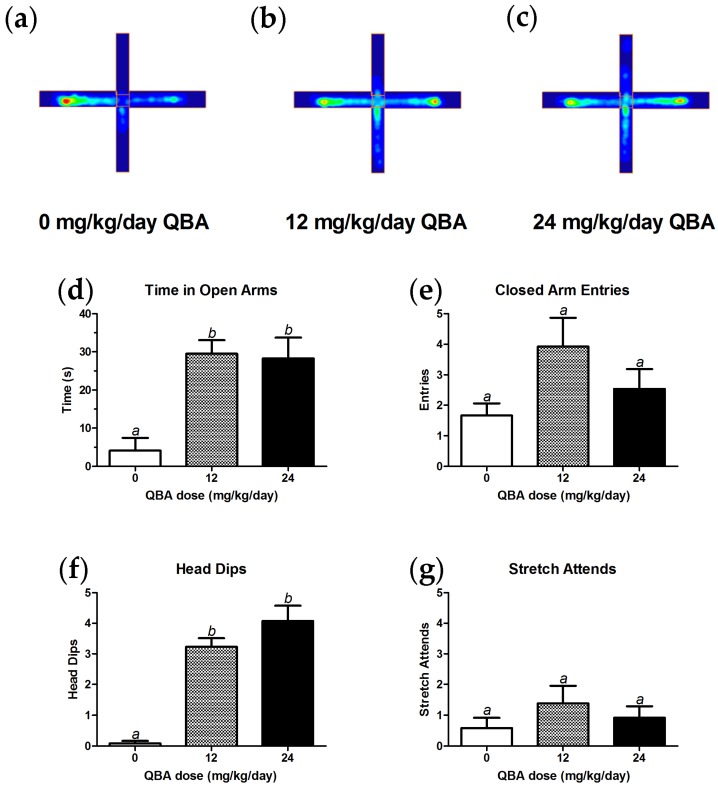
QBA decreased anxiety in aged male rats. Qualitative, positional heatmaps show the average time spent in the open arms (vertical axis) and closed arms (horizontal axis) of the elevated plus maze for (**a**) controls, (**b**) animals given 12 mg/kg/day QBA and (**c**) animals given 24 mg/kg/day QBA (blue = low amount of time; yellow = intermediate; red = high amount of time). Quantitation and analysis of the plus-maze behaviors indicated that QBA increased the amount of time spent in the open arm (**d**), had no effect on the number of closed arm entries (**e**), increased the number of head dips (**f**), and did not affect stretch-attend behavior (**g**). Lack of shared superscripts indicates groups that differed significantly (*p* < 0.05) in Tukey’s post hoc test. *N* = 12–13 for each experimental arm. Error bars represent the SEM.

Anxiolytic in vivo effects of QBA were first noted in the elevated plus maze assay. Heat maps of the amount of time spent in different parts of the plus maze provided qualitative visualization of the behavior of ~17-month-old male rats after consuming different amounts of QBA for ~5 months. Control animals (0 mg/kg/day QBA; Figure 3a) spent less time in the open arms of the maze than animals that were receiving 12 mg/kg/day QBA (Figure 3b) or 24 mg/kg/day QBA (Figure 3c), and all three treatment groups spent equivalent amounts of time in the closed arms of the maze. Quantitation and analysis of these data indicated that the differences in open arm time between the control group and both treatment groups were significant (F_(2,35)_ = 10.99, *p* < 0.001; Figure 3d). In contrast, there were no differences between the 12 and 24 mg/kg/day groups for open arm time, and the number of entries into the closed arms of maze were equivalent amongst all three groups (Figure 3e). Treatment with QBA (12 and 24 mg/kg/day) also significantly increased the number of head dips in comparison to control animals (F_(2,35)_ = 36.69, *p* < 0.001; Figure 3f), but QBA had no effect on stretch-attend behavior (Figure 3g).

The potential effects of QBA on locomotor activity, cognition, and depressive-like behaviors were also evaluated using the same animals in the open field arena, Barnes maze and the forced swim test, respectively. However, no main effects of treatment were detected in the distance traveled or center time in the open field, in task acquisition latency or memory probe in the Barnes maze, nor in immobility, climbing or diving behaviors in the forced swim test (data not shown).

### 3.4. Weight Maintenance

**Figure 4:**
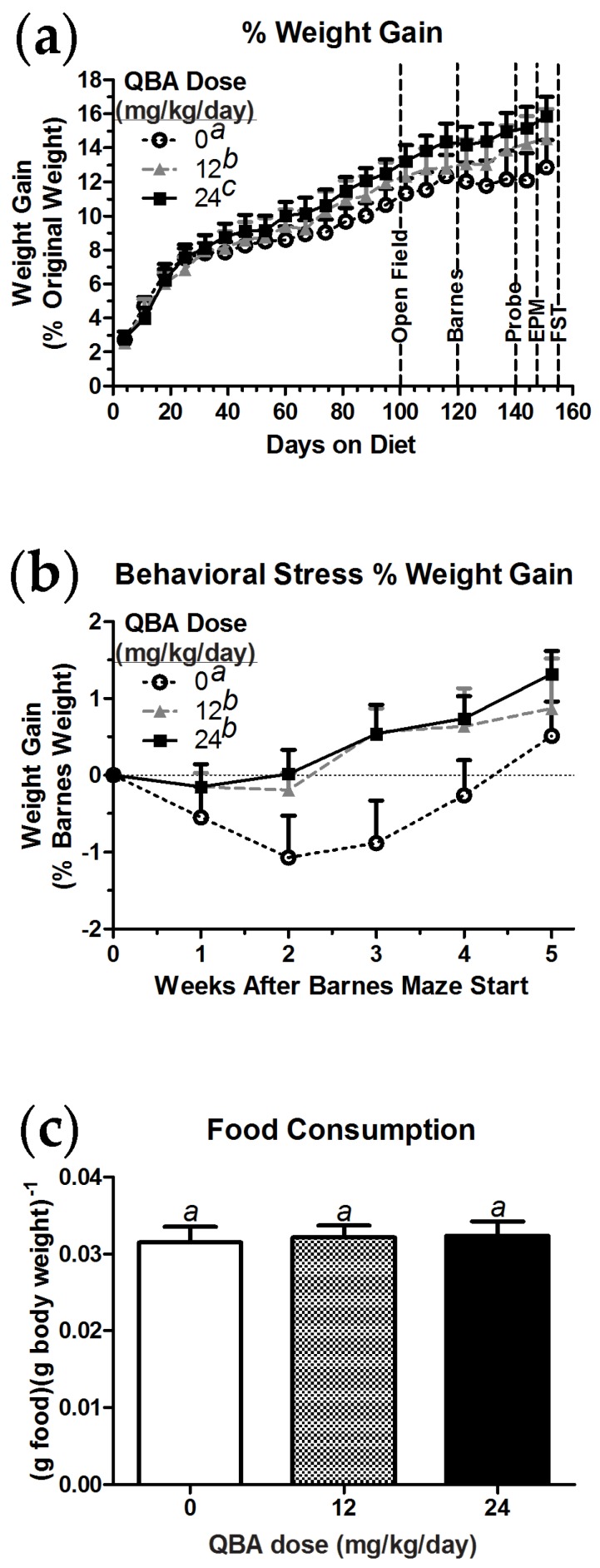
QBA increased the weight of aged male rats and mitigated the weight loss associated with the stress of behavioral assessment. (**a**) All of the animals gained weight as they aged, but QBA-treated animals gained significantly more than controls. The weight-gain differences became more pronounced with the onset of cognitive testing in the Barnes maze at study day 120. (**b**) In comparison to animals that received QBA, the control animals exhibited significant weight loss during behavioral testing in the Barnes and elevated plus mazes. (**c**) The differences in body weight were not due to differences in food consumption. Barnes = Barnes maze task acquisition; Probe = Barnes maze memory probe task; EPM = elevated plus maze testing; FST = forced swim test. Lack of shared superscripts indicates statistically significant differences (*p* < 0.05) in Tukey’s post hoc test. *N* = 11–13 per group. Error bars represent the SEM.

The body weight of the aged male rats was monitored throughout the course of the study as a rough assessment of potential toxicity. As shown in Figure 4a, control animals consistently gained weight as they aged during the pretreatment phase, but their weight gain plateaued once the Barnes maze testing commenced. In contrast, and unexpectedly, animals consuming QBA gained significantly more weight than the control animals (F_(2,42)_ = 33.19, *p* < 0.001), and the animals treated with 24 mg/kg/day QBA gained significantly more weight than the group treated with 12 mg/kg/day QBA. When the data were recalibrated to the animals’ weights at the start of Barnes maze testing (Figure 4b), a significant increase in weight gain was detected in the QBA-treated animals relative to controls (F_(2,10)_ = 11.50, *p* = 0.003). However, the weight gain in the control animals did begin to recover towards the end of the study. All three treatment groups ate equivalent amounts of food (Figure 4c).

### 3.5. Body Composition in Mice

The surprising effects of QBA on weight gain in the aged rats prompted questions of where in the body the weight was being added, whether there were sex effects, and whether the weight gain could be detected in a second species. Mice (BALB/c) were chosen for this second set of experiments because they could address all of the above questions and were more compatible with the body composition instruments that were available. The mice were given dietary QBA doses (0, 30 and 60 mg/kg/day) that were equivalent to the doses provided to the rats, as determined by the body surface area method [28]. The body compositions of the mice were then monitored for four months of QBA exposure.

**Figure 5:**
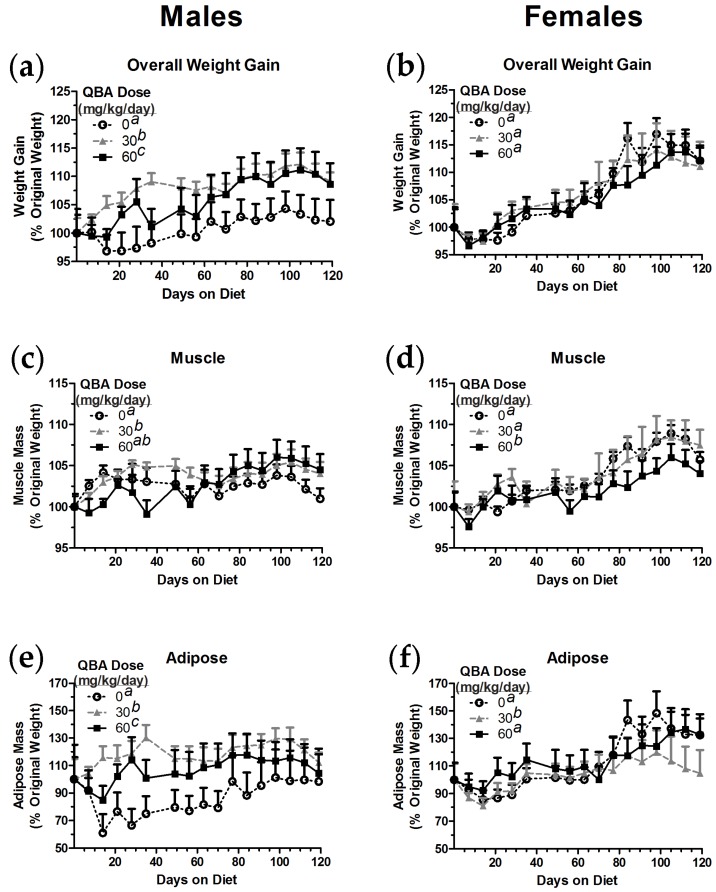
QBA affected the weight gain and body composition of mice in a sex-dependent manner. (**a**) The administration of QBA to male mice at doses and durations that were equivalent to those given to the aged, male rats also resulted in significantly increased weight gain in comparison to control animals. (**b**) In contrast, QBA did not affect weight gain in female mice. (**c**) Muscle mass was significantly increased in male mice by 30 mg/kg/day QBA. (**d**) Muscle mass in female mice was not affected by 30 mg/kg/day QBA, but 60 mg/kg/day QBA significantly reduced muscle mass when compared to controls. (**e**) Significant increases in adipose tissue were associated with QBA treatment in male mice. (**f**) However, female mice receiving 30 mg/kg/day QBA exhibited significant reductions in adipose tissue when compared to controls. Treatment groups not sharing common superscripts differed significantly (*p* < 0.05) in 1-way RMANOVA followed by Tukey’s post hoc tests. *N* = 10–15 per group. Error bars represent the SEM.

QBA affected weight gain in male mice in a manner similar to that observed in male rats. Control male mice gained significantly less weight than male mice given QBA (F_(2,32)_ = 72.79, *p* < 0.001), and, due to increases seen early in the study, the 30 mg/kg/day group gained more weight on average than the 60 mg/kg/day group (Figure 5a). In contrast, QBA did not change overall weight gain in female mice (Figure 5b).

Some of the overall weight gain in male mice was attributable to a significant gain in muscle mass (Figure 5c). Male mice in the 30 mg/kg/day group exhibited significantly greater muscle mass than control animals (F_(2,32)_ = 3.52, *p* = 0.041). Male mice given 60 mg/kg/day QBA trended towards having greater muscle mass, but this group had an intermediate phenotype that was not significantly different from controls or the 30 mg/kg/day group. Female mice that were consuming 60 mg/kg/day QBA had significantly lower muscle mass than female mice in both the control group and the 30 mg/kg/day group (F_(2,32)_ = 16.66, *p* < 0.001; Figure 5d). 

Adipose tissue was also significantly increased by QBA in male mice, but female mice saw a significant decrease in this tissue type with QBA treatment. Both of the doses of QBA increased adipose in male mice (F_(2,32)_ = 60.34, *p* < 0.001), but 30 mg/kg/day was the most effective dose in this context (Figure 5e). Similarly, 30 mg/kg/day QBA was the most effective dose in female mice, but, in contrast to the data observed in the males, QBA significantly reduced female adipose tissue (F_(2,32)_ = 7.37, *p* = 0.002; Figure 5f).

**Figure 6:**
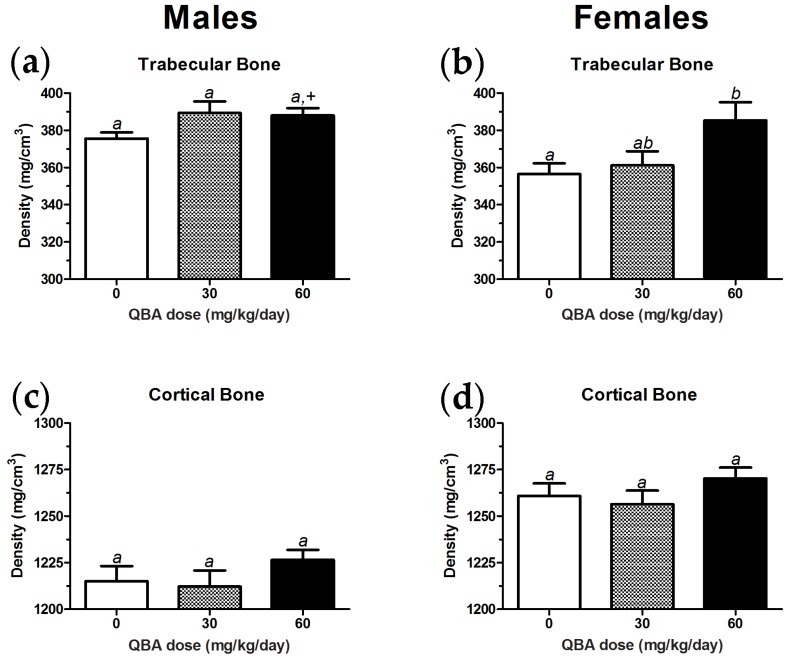
QBA increased trabecular bone density in female mice. (**a**) A non-significant trend for increased trabecular bone density was noted in male mice. Significant differences between the control group and animals given 60 mg/kg/day QBA were only detected when an unpaired *t*-test was used. (**b**) Trabecular bone density in female mice was significantly increased by QBA, but only the 60 mg/kg/day group differed from controls. (**c**,**d**) Cortical bone density was not altered by QBA treatment in either sex. Treatment groups not sharing common superscripts differed significantly (*p* < 0.05) in 1-way ANOVA followed by Tukey’s post hoc tests. +, significantly different from control group in two-tailed, unpaired *t*-test. *N* = 9–12 per group in males and *N* = 7–15 per group in females. Error bars represent the SEM.

Trabecular bone density increased with QBA treatment in female mice, but only an increasing trend was detected in male mice. The ANOVA analysis of trabecular bone density in male mice did not find a significant effect of QBA. However, a two-tailed, unpaired *t*-test comparison of the 60 mg/kg/day group to the controls did reach significance (*t*_(19)_ = 2.29, *p* = 0.034; Figure 6a). A main treatment effect of QBA was found in the trabecular bone density of female mice (F_(2,27)_ = 4.17, *p* = 0.026), and the 60 mg/kg group differed significantly from the controls (Figure 6b). Figure 6c,d shows that cortical bone density was not affected by QBA in either male or female mice, respectively.

## 4. Discussion

The results of the studies reported here clearly demonstrate that QBA has functional benefits for anxiety-related behavior, the viability and growth of hippocampal neurons, and sex-dependent changes in the mass of muscle, bone, and adipose tissue. It is currently unknown if QBA interacts with sex on the behavioral and in vitro measures that were conducted, but sex had significant effects on the detailed body composition analyses in mice. These findings strongly suggest that the QBA effect on body composition may interact with sex hormones. However, although the current literature is suggestive of a relationship between QBA, and, in particular, estradiol, direct experiments of this potential relationship within the contexts of anxiety, neuroprotection, and body composition have not been performed.

Feeding of QBA (12–24 mg/kg/day) for 5 months significantly increased the amount of time spent in the open arm of the elevated plus maze by aged, male rats, indicating that QBA-treated animals had reduced anxiety-related behaviors in these tests. We used aged animals to enable a comparison to the data available from young animals that had been exposed to RJ or QBA. Our findings are similar to those in young, male mice (49–70 days old), in which QBA administration (100–500 µg/kg/day injected intraperitoneally for 21 days; equivalent to ~50–250 µg/kg/day in rats) decreased anxiety-like and depressive-like behaviors when the mice were also exposed to stress. The authors also noted that 2500 mg/kg/day RJ that was provided orally had similar effects, but QBA had no effect in unstressed mice [19]. On one hand, a conservative 2% estimate of the QBA content of the RJ [1,17] in these experiments indicates that the oral RJ provided 50 mg/kg/day of QBA (0.02 × 2500), which is equivalent to the 30–60 mg/kg/day that was provided to the mice and the 12–24 mg/kg/day that was provided to the rats in this study [28]. On the other hand, it can also be argued that mice exposed to daily injections do indeed experience stress as daily saline injections upregulate nicotine-related stress responses in manners that are not different from daily nicotine injections [31]. As such, the reported anxiolytic effects of QBA in mice may have some confounds, but our data from normally-aged, unstressed rats given QBA passively through the diet are generally consistent with the mouse findings.

We did not observe any effects of QBA in the forced swim test. In contrast, 2500 mg/kg/day RJ (~88 mg/kg/day QBA) normalized the immobility time of ovariectomized (OVX) female rats in the forced swim test [32], suggesting that the lack of QBA efficacy in similar experiments in aged males could be due to sex effects or to the lower QBA doses that we used.

Differences in anxiety- or depressive-related behaviors are typically associated with changes in neurotransmitter levels [33,34,35]. Interestingly, eight weeks of RJ (50–100 mg/kg/day; 21–42 mg/kg/day QBA) feeding increased dopamine turnover in the prefrontal cortex of aged, male rats [36], and oral RJ (300 mg/kg/day) for 30 days protected developing rat pups from tartrazine-induced losses of brain GABA, dopamine, serotonin, catalase, superoxide dismutase, and glutathione. Accordingly, RJ also protected the pups from tartrazine-induced increases in malonyldialdehyde, a measure of lipid peroxidation [37]. Similarly, the feeding of RJ to aged, female rats for seven months increased mRNA expression for thyroid stimulating hormone-β (TSH-β), suggesting that RJ countered age-related decreases in pituitary function [38]. Although QBA was not tested directly in these neurotransmitter studies, their findings suggest that similar neuroprotective effects of QBA may have occurred in our experiments. 

The viability, mitochondrial health and growth of primary hippocampal neurons were supported by QBA. In our models of age-related neurodegeneration (glutamate challenge) and stroke (hypoxia challenge), QBA-treated neurons exhibited reduced cell death and increases in properly polarized mitochondria when compared to untreated controls. Neurons receiving QBA also covered a significantly larger area than control neurons did, indicating that QBA-treated neurons grew larger and likely made more interconnections with one another. These growth effects were consistent with earlier work, in which RJ stimulated neural stem cells to differentiate into neurons, astrocytes, and oligodendrocytes, but QBA (1–100 µM) stimulated neuronal differentiation while inhibiting astrocyte differentiation [18]. These in vitro growth and differentiation effects of RJ/QBA may translate in vivo. Mice fed 1% RJ (~1.5 g RJ/kg; ~3 mg QBA/kg) for 10 days exhibited increased mRNA for glial-derived neurotrophic factor (GDNF) and neurofilament H in the hippocampus, measures that are consistent with the differentiation and growth of astrocytes and neurons, respectively. Although QBA was implicated as a causal factor in these experiments due to the higher likelihood that it would cross the blood-brain barrier, QBA alone was not tested directly [9].

The mechanisms through which QBA exerts its effects in the brain appear to be through pro-growth signaling. RJ stimulates the phosphorylation and activation of erk-1, erk-2 and cyclic adenosine monophosphate response element binding (CREB) protein in neural stem cells. Yet, another RJ component, adenosine monophosphate (AMP)-N1 oxide, acted through adenosine receptors and integrin signaling to promote neurite outgrowth, and RJ containing QBA was not superior to AMP-N1 oxide alone [39]. Other QBA-like compounds, such as trans-2-decenoic acid ethyl ester, activate erk-related BDNF expression in neurons, but BDNF receptors are not needed for erk activation by these compounds [20]. Despite some open questions as to the exact mechanism of QBA effects in neurons, erk activation would explain the increases in neuron growth and mitochondrial health that we observed. Signaling by erk drives the expression of syntaxin, synapsin, synaptophysin, and BDNF, which are needed for the growth of neuronal synaptic connections and for neuronal survival [20]. Similarly, erk activation increases mitochondrial biogenesis that results in better calcium buffering and neuroprotection during hypoxia [40], which aligns with our observations of increased TMRE signals in neurons when protected by QBA. It was also speculated that QBA could induce neurite outgrowth by activating collagen production and subsequent integrin signaling [39], but this assertion was based on QBA effects in fibroblasts [41], not neurons. 

The finding that QBA increases collagen production in fibroblasts [41] suggests an interaction with the estradiol system, as estradiol has the same effect [42]. Estradiol also increases neurite outgrowth [43], protects neurons from stress-related death [44], improves mood in a dose-dependent manner [45,46,47,48], and activates erk through non-genomic mechanisms [23]. Royal jelly has activity at estradiol receptors, yet its estrogenic activity may not cross the blood-brain barrier, as evidenced by RJ’s ability to induce vascular endothelial growth factor (VEGF) in the uterus but not the brain. However, these were acute experiments that ended 6 h after a single, subcutaneous injection of RJ (1 g/kg) [13], not after prolonged dietary exposure.

In breast cancer cell lines, QBA (100 pM–10 µM) modulates estrogen receptor function by binding to an allosteric pocket on the receptors, but it does not compete with estradiol binding [21,22]. QBA (1 µM) alone increased recruitment of estrogen receptor-β (ERβ) to its promoter compared to controls, but QBA was less effective in these measures than estradiol alone (10 nM). The combination of QBA + estradiol also increased the recruitment of ERα and ERβ when compared to controls, but, again, the combination was significantly less effective than estradiol alone [21]. The apparent antagonism of ER activity by QBA in these experiments could have been due to the high concentration of estradiol used or its combination with QBA, since the QBA-alone data indicated that QBA was an ER agonist. Such circumstances are possible because circulating levels of estradiol are typically in the picomolar range [48,49], and high agonist concentrations can result in inverted, U-shaped concentration-response curves [50,51]. Similarly, as reported here, our in vitro experiments of QBA effects on neurite extension and on mitochondrial health after hypoxia challenge showed that higher concentrations are not always better. Overall, these results suggest that QBA is a modulator of estradiol receptors and can have estrogenic effects in the absence of estradiol or at the lower levels observed in males [52]. By extension, these findings provide insights towards the sex differences in the body composition results, as well as some support for QBA in menopause-related issues.

In comparison to controls, aged male rats that consumed QBA exhibited increased weight gain and better weight maintenance during behavioral stress. The likely distribution of this weight was determined by body composition analyses of male and female mice during their exposure to experimental conditions that delivered QBA doses that were equivalent to those of the male rats. Male mice that consumed QBA gained weight in a fashion that was similar to that of QBA-treated male rats, while QBA-treated female mice did not gain weight in comparison to control females. Interestingly, QBA-treated female mice demonstrated an increase in bone density. None of the differences in weight gain were due to differences in diet consumption, as all of the treatment groups consumed equivalent amounts of food. These findings are consistent with those obtained from OVX female rats that had received RJ treatments: as compared to controls, there were no changes in body weight, but bone mineral density was increased [53]. These effects, like ours, were most notable in the trabecular bone. Unfortunately, Hidaka et al. (2006) [53] did not conduct similar in vivo experiments in normal animals. However, ex vivo experiments in which bone tissue was cultured from normal rats showed that RJ increased calcium content without affecting bone resorption by osteoclasts [53].

As observed in neurons, the effects of RJ and QBA on bone-related cells have some basis in the estrogen receptor system, and higher doses are not always better. Osteoblast-like MC3T3 cells have been stimulated by RJ (0.1–1 mg/mL) to express more collagen than controls, and 0.1 mg/mL RJ was more effective than 1 mg/mL RJ in these experiments. The effects of RJ on increasing the proliferation of MC3T3 cells were also more effective at lower concentrations, and both effects were inhibited by an estrogen-receptor antagonist [54]. Interestingly, QBA alone (1–10 nM) increased mineralization in osteoblasts to an extent that was equivalent to estradiol (1 nM). Higher concentrations of estradiol (10–100 nM) were more effective than QBA in these mineralization assays, but the highest tested concentrations of estradiol (1 µM) or QBA (100 nM) were not different from controls [21]. Lastly, oral administration of RJ increased the expression of procollagen I mRNA [54], as well as the proliferation of osteoblasts, osteoclasts and blood vessels in a rat model of bone remodeling [55]. Therefore, the increased bone density in female mice we observed with QBA is consistent with the RJ literature; suggesting that QBA is the active component of RJ that increases bone density by increasing bone-cell proliferation, collagen production and mineralization through mechanisms that are related to estrogen receptors. 

Our findings in adipose and muscle tissue suggest again that QBA shares signaling properties with estradiol. Adipose mass was increased by QBA (30 and 60 mg/kg/day) in males, but QBA (30 mg/kg/day only) decreased adipose tissue in females. Similarly, muscle mass was increased by QBA (30 mg/kg/day only) in males, but QBA (60 mg/kg/day only) decreased muscle mass in females. These gross actions of QBA appear consistent with the associations between estradiol, loss of lean mass [56], and muscle protection [57] in human males as well as between estradiol, adiposity, and glucose utilization in human females [58]. The presence of ERα and ERβ in both adipose and muscle tissue, as well as their expression ratio, may have some bearing on the ultimate effects of estradiol in these tissues [59,60,61]. In addition, estrogen receptors are differentially expressed in males and females, depending on the circulating levels of endogenous sex hormones (e.g., testosterone and estradiol as well as testosterone being converted to estradiol by aromatase, even in the brain; reviewed by Handa and Weiser, 2014) [62]. These circumstances also provide some explanations for the sex differences and dosing differences that are similar to the paradoxical QBA-estradiol interactions in breast cancer cells [42] and osteoblasts [48]. In contrast, sex differences cannot be expected in our in vitro experiments because the hippocampal cultures were derived from E17–18 rat pup brains. Embryonic day 17–18 is too early to sex the pups, and cultures made from later time points are not optimal [22]. In addition, E17–18 is prior to the testosterone surge that masculinizes the brain at postnatal day 1 [63]. Thus, the hippocampal neurons used for the current work expressed estrogen receptors, but there was no reason to expect sex differences at the stage of development from which they were derived. 

Other potential mechanisms for the action of QBA may exist. In vitro administration of RJ to myotubes increased their diameter, proliferation, and expression levels of insulin growth factor-1 (IGF-1) receptors and activated Akt. When RJ was then provided to aged, male mice, it increased muscle mass, the duration of hang time in the grip test, and the regeneration of muscle tissue after injury. These in vivo benefits were also associated with the increased expression of serum IGF-1 [64]. When RJ was provided to female mice for 12 weeks, increases in the expression of adiponectin and activation of 5′-AMP-activated protein kinase (AMPK) were observed [65]. Unfortunately, the amounts of QBA in these two RJ experiments were unknown. Nonetheless, dietary restriction reduces bodyweight and activates AMPK, usually as a result of the changes in the AMP: ATP ratio, which is a function of cellular energy availability. However, QBA (20–25 µM in vitro; 300 mg/kg in vivo) upregulated the expression and activity of AMPK in muscle cells in a manner that was independent of the AMP: ATP ratio [66]. Mechanisms of QBA that involve cytokine induction [67,68] or inhibition of histone deacetylases [2] have also been suggested, but the effective QBA concentrations in these experiments were in the millimolar range. Overall, the only mechanisms of QBA that are likely to be engaged under the conditions of our experiments (12–60 mg/kg/day depending on species; 0.1–30 µM in vitro) are those that involve estrogen receptors and erk signaling that could activate AMPK. Alternatively, given that the estrogen receptor activity increases erk signaling [23], and erk has been connected to BDNF expression [20] as well as AMPK through liver kinase B1 [69], it could be speculated that all of the actions of QBA are mediated by QBA-estrogen receptor interactions. Such assertions are based on findings in cancer cell lines [21,22] and require substantiation in the target tissues, but the sex-dependent effects on body composition in the current work do align with explanations that involve estrogen receptors.

## 5. Conclusions

The experiments that are reported here strongly indicate that QBA is an active component of RJ that increases neuron growth, protects neurons from damage, and decreases anxiety-like behavior. Furthermore, QBA has sex-dependent benefits for bone density, adiposity and muscle mass. The most likely mechanisms for these benefits are through regulation of estrogen receptors and subsesquent engagement of erk-related signaling, but these associations have not been substantiated directly in the specific systems that they might involve. Future experiments should seek to address these mechanistic possibilities and how they relate directly to the in vivo effects of QBA.
